# Genome wide identification of the *NPR1* gene family in plant defense mechanisms against biotic stress in chili (*Capsicum annuum* L.)

**DOI:** 10.3389/fmicb.2024.1437553

**Published:** 2024-08-05

**Authors:** Qandeel Ishfaqe, Adnan Sami, Muhammad Zeshan Haider, Arsalan Ahmad, Muhammad Shafiq, Qurban Ali, Alia Batool, Muhammad Saleem Haider, Daoud Ali, Saud Alarifi, Md Samiul Islam, Muhammad Aamir Manzoor

**Affiliations:** ^1^Department of Plant Pathology, Faculty of Agricultural Sciences, University of the Punjab, Lahore, Pakistan; ^2^Department of Plant Breeding and Genetics, Faculty of Agricultural Sciences, University of the Punjab, Lahore, Pakistan; ^3^Department of Entomology, Faculty of Agricultural Sciences, University of the Punjab, Lahore, Pakistan; ^4^Department of Zoology College of Science King Saud University, Riyadh, Saudi Arabia; ^5^Graduate School of Agriculture, Hokkaido University/Bioproduction Research Institute, National Institute of Advanced Industrial Science and Technology, Sapporo, Japan; ^6^Department of Plant Science, School of Agriculture and Biology, Shanghai Jiao Tong University, Shanghai, China

**Keywords:** *NPR1*, geminivirus, begomovirus, chili pepper, biotic stress, virus titer

## Abstract

Chili pepper cultivation in the Indian subcontinent is severely affected by viral diseases, prompting the need for environmentally friendly disease control methods. To achieve this, it is essential to understand the molecular mechanisms of viral resistance in chili pepper. The NONEXPRESSOR OF PATHOGENESIS-RELATED GENES 1 (*NPR1*) genes are known to provide broad-spectrum resistance to various phytopathogens by activating systemic acquired resistance (SAR). An in-depth understanding of *NPR1* gene expression during begomovirus infection and its correlation with different biochemical and physiological parameters is crucial for enhancing resistance against begomoviruses in chili pepper. Nevertheless, limited information on chili *CaNPR* genes and their role in biotic stress constrains their potential in breeding for biotic stress resistance. By employing bioinformatics for genome mining, we identify 5 *CaNPR* genes in chili. The promoter regions of 1,500 bp of *CaNPR* genes contained cis-elements associated with biotic stress responses, signifying their involvement in biotic stress responses. Furthermore, these gene promoters harbored components linked to light, development, and hormone responsiveness, suggesting their roles in plant hormone responses and development. MicroRNAs played a vital role in regulating these five *CaNPR* genes, highlighting their significance in the regulation of chili genes. Inoculation with the begomovirus “cotton leaf curl Khokhran virus (CLCuKV)” had a detrimental effect on chili plant growth, resulting in stunted development, fibrous roots, and evident virus symptoms. The qRT-PCR analysis of two local chili varieties inoculated with CLCuKV, one resistant (V1) and the other susceptible (V2) to begomoviruses, indicated that *CaNPR1* likely provides extended resistance and plays a role in chili plant defense mechanisms, while the remaining genes are activated during the early stages of infection. These findings shed light on the function of chili’s *CaNPR* in biotic stress responses and identify potential genes for biotic stress-resistant breeding. However, further research, including gene cloning and functional analysis, is needed to confirm the role of these genes in various physiological and biological processes. This in-silico analysis enhances our genome-wide understanding of how chili *CaNPR* genes respond during begomovirus infection.

## Introduction

*NPR1* (NON-EXPRESSOR OF PATHOGENESIS-RELATED GENES-1) gene family members are regulated by salicylic acid signaling pathways and play a crucial role in disease resistance and immunity in plants (Backer et al., 2019). Horticultural crops need to have robust defense and immune systems against disease and pest assault since they are vulnerable to many pathogens, including viruses (Thulasi Devendrakumar et al., 2019). *NPR1*-based resistance has been employed in many crops to control pathogen infection (Liu et al., 2002; Silva et al., 2018).

The *NPR1* gene family, initially characterized in *Arabidopsis thaliana*, has been extensively studied for its role in modulating immune responses through the salicylic acid pathway (Shi et al., 2010). Studies in Arabidopsis have provided foundational insights into the molecular mechanisms and pathways involved, which are now being translated to other plant species, including horticultural crops (Wong et al., 2002). The selected *NPR1* genes in our study have been thoroughly investigated in *Arabidopsis thaliana*, and these findings are being applied to understand and enhance disease resistance in a variety of horticultural crops (Zhang et al., 2024).

NPR1 plays a pivotal role in bolstering the immune system of plants and serves as the primary regulator of salicylic acid, a crucial plant hormone. *NPR1’s* involvement in plant defense includes binding to the promoters of PR genes to activate their expression (Wang et al., 2016). Within the nucleus, *NPR1* collaborates with transcription factors to stimulate PR gene expression, thereby initiating the plant’s defense mechanisms. The expression of *NPR1* is a key trigger for enhancing the plant’s immune response. In particular, salicylic acid (SA) facilitates NPR1’s interaction with two vital proteins, CDK8 (CYCLIN-DEPENDENT KINASE-8) and WRKY18 (WRKY DNA-BINDING PROTEIN-18), especially in *Arabidopsis* (Chen et al., 2020b). The multifaceted roles of NPR1-like proteins underscore their importance in a complex family that contributes significantly to both plant immune responses and growth processes (Jin et al., 2018).

Overexpressing the *NPR1* gene in *Arabidopsis thaliana* enhances resistance against bacterial pathogens such as *Pseudomonas syringae* and *Xanthomonas campestris*, and oomycete pathogens like *Phytophthora infestans* (causing late blight in potato and tomato) and *Pythium* species (causing damping-off disease in various plants; Wally et al., 2009). Both *NPR1* and *PR* genes play a fundamental role in a plant’s response to pathogen challenges. *NPR1* is a key player in establishing Systemic Acquired Resistance (SAR) and Induced Systemic Resistance (ISR). SAR is an inducible plant defense mechanism that provides broad-spectrum immunity against secondary infections in plant tissues beyond the initial infection site, with *NPR1* serving as a crucial regulator (Zhang et al., 1999). When the *NPR1* gene is obstructed, either through proteasome inhibitors or genetic manipulation of Cullin-3, it activates the expression of *NPR1* target genes in uninduced cells, albeit to a lesser degree than salicylic acid treatment. Importantly, the degradation of *NPR1* is not prevented by salicylic acid treatment or SAR activation triggered by pathogens (Wang et al., 2021). Plants guard themselves by introducing local and systemic defense responses against pathogens (Li et al., 2020). *NPR1* plays a vital role in the salicylic acid (SA)-mediated systemic acquired resistance (SAR) pathway (Backer et al., 2015); in the establishment and control of systemic acquired resistance (SAR) as well as induced systemic resistance (ISR) which can restrict the spread of virulent pathogen infections in plants. *NPR1* modulates cross-talk between SA and JA signaling pathways (Wang et al., 2020). SA induces plant defense and immunity in the *C. sativus*. SA-SA-mediated defense response gives protection against biotrophic and hemi-biotrophic pathogens, whereas JA-mediated defense response against many necrotrophic pathogens (Backer et al., 2019).

Chili (*Capsicum* spp.) is an important commercial crop that is grown all over the world. It is a dicotyledonous flowering plant and belongs to the family *Solanaceae* with different names such as hot pepper, chili pepper bell pepper etc. having superfluous nutritional and medicinal value (Knapp et al., 2004). Pakistan is among the main red chili producing countries of the world, exporting 2,817 metric tons of whole and powdered red chili worth $5,727,738 (PKR 916.44 million) in 2018–2019. As a high-value cash crop with an average net income of $2,344 (PKR 375,000) per hectare, red chili peppers are a lucrative crop for smallholder farmers (Pakistan Agriculture Development (PAD)). In 2013, global production of chili pepper (both green and dried) was 34.6 million tonnes with 47% of output coming from China alone. As per the data of (Bhatti et al., 2024) India, Bangladesh, Ethiopia, Thailand, and China are among the world’s top producers of chili peppers while Pakistan stand at 6th rank with total production of 144.2 M Kg. The Chili genome is a representative complex plant genome; it has one of the largest genome sizes in the *Solanaceae* family at ~3.5GB and is comprised largely of repetitive elements, estimated at 75–80% of the genome (Hulse-Kemp et al., 2018). The hot pepper genome (Mexican landrace of *Capsicum annuum* cv. CM334) has been sequenced by an international group of scientists from Korea, Israel, and USA. The sequence was published in Nature Genetics in January 2014 (Kim et al., 2014).

Pathogen sensitivity in chili cultivation is a critical concern for farmers, as these vibrant and spicy peppers are susceptible to a range of diseases that can devastate their yields (Singh, 2013). Several pathogens can affect chili crops, posing a significant threat to both the quality and quantity of the harvest (Shah and Smith, 2020). Begomovirus belongs to geminiviridae family of viruses that infect chili plants, causing diseases like leaf curl and yellowing (Maurya et al., 2019). They are characterized by circular single-stranded DNA genomes and are transmitted by whiteflies, posing a significant threat to chili cultivation worldwide (Mansoor et al., 2003). Other than begomoviruses, viruses from different families and genera such as Tobacco mosaic virus (TMV; Ontañón et al., 2024) and Cucumber mosaic virus (CMV; Ahmed et al., 2024) can also infect chili plants, causing symptoms such as mosaic patterns and stunted growth (Ala-Poikela et al., 2005). Among the other pathogens that target chili plants are bacterial blight (Pawaskar and Kerkar, 2021; caused by *Xanthomonas campestris pv. vesicatoria*), powdery mildew (García-Gaytán et al., 2016; resulting from the fungus *Leveillula taurica*), and *Phytophthora capsici*, (Sanogo, 2004) which leads to root rot and fruit rot (Xie et al., 1999). Additionally, fungal pathogens like Fusarium and Verticillium (Sanogo and Carpenter, 2006) species can also attack the plant, leading to wilting and reduced fruit production. The qRT-PCR assessment of two local chili varieties, one resistant (V1) and other prone to geminivirus (V2), suggested that *CaNPR1* likely extends resistance and contributes to chili’s defense mechanisms, while the other genes are activated during the initial infection stages. These findings provide insights into the role of *CaNPR* genes in chili’s response to biotic stress and pinpoint potential genes for breeding biotic stress-resistant varieties. However, further research, including gene cloning and functional analysis, is essential to validate these genes’ roles in various physiological and biological processes. This in-silico analysis enriches our comprehensive knowledge of chili *CaNPR* genes at the genome-wide level. To address the challenges of gene transformation in chili for biotic resistance, tissue culture techniques can be employed to ensure stable integration of *NPR1* genes into the plant genome. Additionally, backcrossing, a natural breeding method, can be used to introduce *NPR1* genes into existing chili varieties while preserving their desirable traits. The objective of this approach is to cultivate biotic-resistant chili varieties through targeted genetic modifications, with success contingent upon the unique traits of the chili variety and the prevailing local environmental conditions.

## Materials and methods

### Identification and phylogenetic analysis of *NPR1* family members in *Capsicum annuum*

Five *NPR1* genes in the *Capsicum annuum* genome were identified^1^ using BLASTP, with *Arabidopsis* BTB domain sequence as query, retrieved from the NCBI.^2^ To confirm the presence of NPR1 like domains, retrieved amino acid sequences were subjected to searches at the SMART (http://smart.embl-heidelberg.de/; Letunic et al., 2006), and NCBI CDD (Conserved Domain Database; https://www.ncbi.nlm.nih.gov/Structure/cdd/cdd.shtml; Marchler-Bauer et al., 2013) with the default parameters. For the phylogeny and sequence comparison 100 *NPR1* genes were also identified from 12 different plants; *Arabidopsis thaliana* (6), *Solanum tuberosum* (6), *Solanum lycopersicum* (6), *Brassica Rapa* (12), *Carica papaya* (4), *Glycine max* (11), *Gossypium hirsutum* (14), *Oryza sativa* (9), *Vitis vinifera* (1), *Triticum aestivum* (20), *Zea mays* (6) and *Capsicum annuum* (5). The *C. annuum* genes *CaNPR* were designated as *CaNPR1* to *CaNPR5*. The multiple peptide sequence alignment of all putative *CaNPR* genes were carried out by MUSCLE using MEGA (7.0) with default options. The phylogenetic trees were constructed using the consequential alignment in MEGA (7.0) following the maximum likelihood (ML) method, and 1,000 bootstrap replications were set for the reliability of the resultant tree.

### Physicochemical properties and subcellular localization

Five *NPR1* genes information was gathered from the Protparam^3^ and Ensembl Plants^4^ databases. Ensembl’s database provided crucial data such as chromosome number, location, and direction, along with mRNA and peptide length. On the other hand, Protparam’s database included information regarding the predicted pI (isoelectric point) molecular weight GRAVY (Grand average of hydropathicity), and Instability index. The WolfPsort database^5^ allowed for the identification of the subcellular localization of the chili proteins. Protein sequences are the input for this database, which uses them to provide a list of probable sites and the prediction scores that go with them. The objective of this study was to identify the likely location of the proteins within the cell (Kavas et al., 2023).

### Cis-regulatory element of *CaNPRs*

Promoter regions were extracted from the Ensembl database (see foot note 4) and subsequently analyzed using the online tool PlantCare^7^ to identify cis-regulatory elements. The system could effectively pull out putative cis-elements from within a range of 5 and 20 bp in the promoters section. A heatmap showcasing these findings was then created using TB-tools (Bülow and Hehl, 2016).

### Conserved motifs and domain analysis of *CaNPRs*

The conserved motif scanning of *CaNPR1* proteins was performed using MEME Suit^8^ and the parameter setting was employed as 50 ≤ width with a maximum number of 20 motifs (Bailey et al., 2015).

### Intron-exon structure analysis of *CaNPRs*

The genomic and CDS sequences of the *CaNPR1* gene family were submitted to the online tool Gene Structure Display Server (GSDS) at http://gsds.cbi.pku.edu.cn/ to examine the distribution of exons and introns (Barre et al., 2009).

### MiRNA analysis for *CaNPR*

The NPR1 gene family of chili was utilized to locate the target location using the PmiREN website.^9^ The CDS sequences of the genes were compared with the mature miRNA sequences using the default parameters of the psRNATarget^10^ web server tool. The relationships between the predicted miRNA and the targeted genes were visualized using the Cytoscape^11^ programme (Mazhar et al., 2023).

### Evolutionary and gene duplication analysis

Deviation time of *CaNPR1* genes was calculated using Ks and Ka values. TB-tool was utilized to estimate the non-synonymous substitution rate (Ka) and synonymous substitution rate (Ks) and also the ratio between them, i.e., Ka/Ks (Chen et al., 2020a). For Ka/Ks calculations the parameters were configured as described in the software package manuals. The molecular evolution of each gene pair was predicted by the Ka/Ks ratios. In general, Ka / Ks <1 means the purification selection, Ka/Ks =1 means the neutral selection, & Ka/Ks >1 means the positive selection (Librado and Rozas, 2009; Rozas, 2009). Deviation time (T) was determined by putting Ks value in T = Ks/2λ equation where λ symbolizes the value of 7.85 × 10^−9^ (Kim et al., 2014).

Dual synteny analysis was performed to address the gene duplication connection in different plants. To check the gene duplication events between different plant species a of plants were selected including *A. thaliana*, *S. tuberosum*, *S. lycopersicum*, *B. Rapa*, *C. papaya*, *G. max*, *G. hirsutum*, *O. sativa*, *V. vinifera* and *Z. mays*. Scan toolkit (MCScanX) in TB-tool was adopted to analyze the gene duplication events, with the default parameters (Wang Y. et al., 2013). The connection between the orthologous of *CaNPR1* genes in other plants was shown by constructing syntenic analysis maps (Chen et al., 2020a).

### Gene ontology of *CaNPR*

A Gene Ontology (GO) analysis was performed using GO annotations to assess the functions of *NPR1* genes in chili. The molecular activities and diverse biological processes of *NPR1* genes were retrived using information sourced from the online database Uniprot (https://www.uniprot.org/; Langenbacher et al., 2020). The *NPR1* gene sequences were then fed into the ShinyGo v0.741 online tool, a resource available at http://bioinformatics.sdstate.edu/go/, to perform the GO enrichment analysis.

### Protein–protein interaction

The verification of the protein interaction involving *NPR1* genes was extended by research. The web-based software known as String database v0.741^12^ facilitated in demonstrating the interaction between proteins within chili’s NPR1 genes.

### Chili cultivars and Geminivirus stress

Locally available seeds of two Chili pepper cultivars V1 (resistant) and V2 (susceptible) were grown in a controlled environment of maintaining optimal temperature, humidity (60–70% RH), and a 12-h photoperiod for chili pepper cultivars at the Department of Plant Pathology, University of the Punjab, Lahore. Total 600 seeds were sown of both varieties, and after 10–12 days’ plants were sorted out based on uniformity in size and health for inoculation. The 60 plants of each variety were divided into 2 treatments with three replicates and agro-infiltration of cotton leaf curl Khokhran virus (CLCuKV) clone was done using a needless syringe. The young leaf samples were collected from control and inoculated plants after the 10 days upon slight appearance of symptoms. The collected samples were immediately placed in liquid nitrogen and stored at -80°C.

### RNA extraction and qRT-PCR analysis

Total RNA was isolated from the young leaf tissues using Pure Link RNA Mini Kit by Invitrogen (Catalogue No. 12183018A). RNA samples were quantified and equalized by Nanodrop Quantification. RNA was then reverse-transcribed to prepare the cDNA using Revert Aid First Strand cDNA Synthesis Kit (Catalogue No. K1622) by Thermo Fisher Scientific by using minimum input of 10 ng/ul of RNA. The primers of 5 *CaNPR1* genes were designed using NCBI Primer-BLAST^13^ list is provided in Table 1. Real Time Expression of targets was performed using SYBR Select Master Mix (Catalogue No.4472903) by taking cDNA as a template with relevant primers. The expression level was measured by 2∆∆CT values against control; GAPDH was used as an internal control. The sets of primers and protocols used for virus titer quantification were followed (Shafiq et al., 2017).

**Table 1:** List of primer used during qRT-PCR.

Gene	Forward/Left	Reverse/Right
*CaNPR1*	GGTGCACCGGTGTATTTTGT	TGCTGGCCTACAAGCTACAT
*CaNPR2*	TGTTCTCATGATGCTTGCGG	TCTGGATGCAGTGCTCAAGA
*CaNPR3*	TGAAGCAGTTCCCTCCAGAG	TGGCAATGGAAAGCAACTCC
*CaNPR4*	GCTAAGAGCGCGCTACATTT	AATCCGCGGCTATGGTGATA
*CaNPR5*	AGCTATACCGGGACATGCTC	TCCAATGTTGTTGCCACCAG

### Statistical analysis

Three independent biological replicates were used for the qRT-PCR analysis, and data was subjected to analysis of variance (ANOVA) using the SPSS Ver. 20 (Chicago, IL, USA). The means of gene expression values were compared using Duncan’s Multiple Range (DMR) test for which the value of *p* < 0.05 was deliberated as statistically significant.

## Results

### Genome-wide identification and characteristic analysis of *CaNPR1* genes and subcellular localization

In this study, we successfully identified five 5 genes with the *C. anuum* genome that encode *CaNPR1* (see foot note 1) using the *Arabidopsis* BTB sequence as query. The mRNA lengths varied from 1749 nt to 1,446 nt, spanning from *CaNPR1* to *CaNPR5*, while the amino acid lengths ranged from 582 bp to 481 bp. The molecular weights (Mw) fell in the range of 6.15 to 5.79 KD (kilo-dalton), with the highest GRAVY value being −0.280 and the lowest −0.140, and the instability index ranged from 52.79 to 36.09 (Table 2).

**Table 2:** Physiochemical properties of NPR1 gene family.

Gene	Accession Number		Chr no.	Location	Orientaion	AA	Length of mRNA	Intron	Mw (KD)	pI	GRAVY	Instability index
	Endemble	PepperGenome										
CaNPR1	PHT76229	Capana07g000893	7	182,569,015–182,590,703	Reverse	582	1749	3	6.09	65129.11	−0.217	48.72
CaNPR2	PHT75980	Capana07g001045	7	124,314,341–124,315,793	Forward	581	1746	3	5.79	65093.47	−0.28	43.15
CaNPR3	PHT62688	Capana02g001303	2	681,563–682,520	Forward	578	1737	3	6.03	64370.58	−0.256	36.09
CaNPR4	PHT71464	Capana10g002328	10	231,735,414–231,736,234	Reverse	491	1,476	1	Not Found	Not Found	−0.248	51.25
CaNPR5	PHT80582	Capana05g000024	5	668,075–669,185	Reverse	481	1,446	1	6.15	52613.83	−0.14	52.79

AA, Amino acid; Mw, Molecular weight; pI, Isoelectric point.

**Figure 1:**
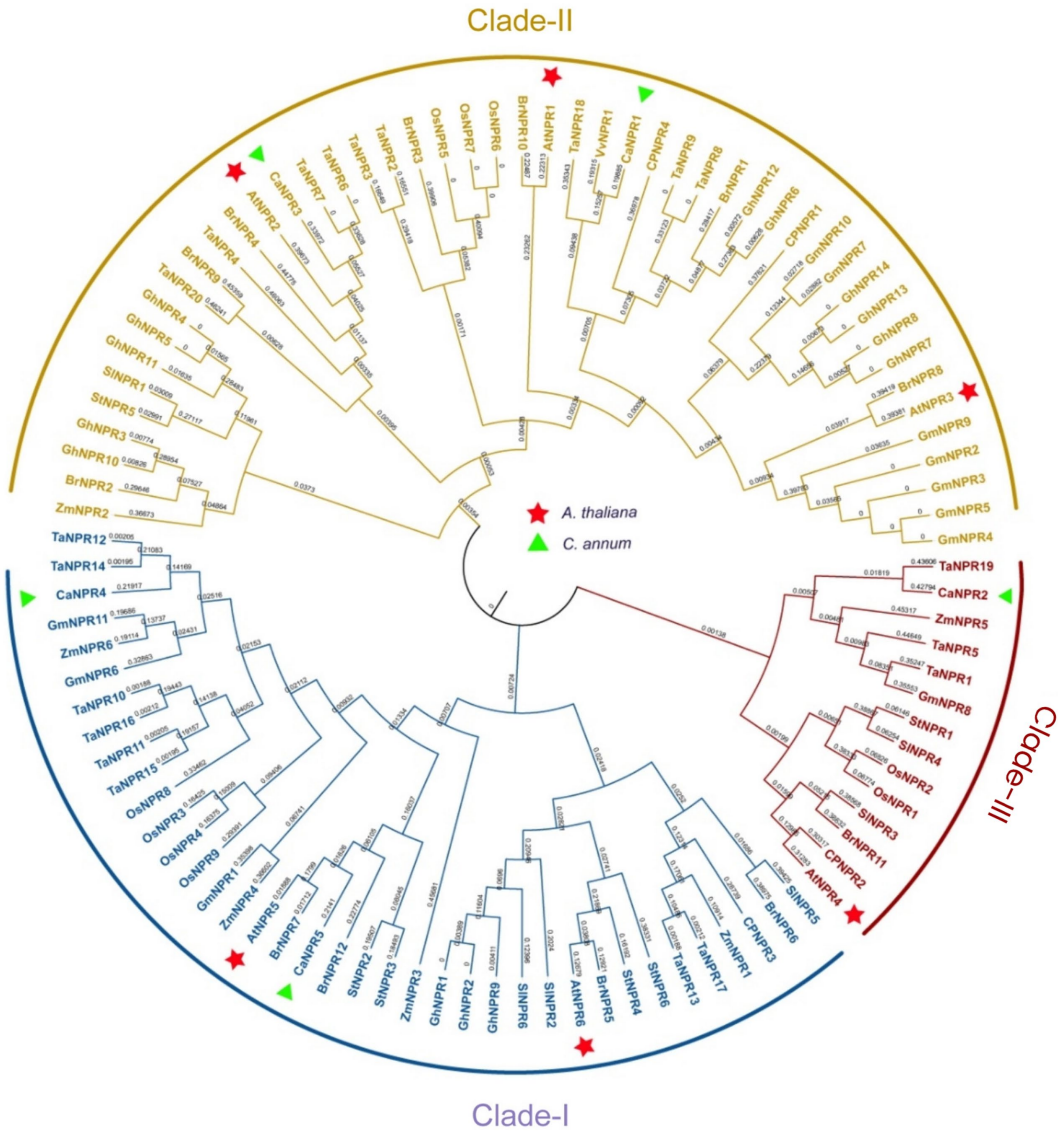
A phylogenetic analysis was conducted on NPR1 homolog proteins derived from various plant species, and the resulting tree was constructed using the maximum likelihood (ML) algorithm in MEGA v7.0. The study involved NPR1-related proteins from 10 different plant species, namely *A. thaliana* (At), *S. tuberosum* (St), *S. lycopersicum* (Sl), *B. Rapa* (Br), *C. papaya* (Cp), *T. aestivum* (Ta), *G. max* (Gm), *G, hirsutum* (Gh), *O. sativa* (Os), *V. vinifera* (Vv), and *Z. mays* (Zm). In this analysis, the *C. annuum* genes, known as *CaNPR*, were specifically designated as *CaNPR1* through *CaNPR5*. The study’s results identified three distinct clades, each represented by a unique color, and the NPRs from *C. annuum* and *Arabidopsis* were differentiated by green and red markers, respectively.

The evolutionary relationship between *NPR1* of *C. annuum*, *A. thaliana*, *S. tuberosum*, *S. lycopersicum*, *B. Rapa*, *C. papaya*, *G. max*, *G. hirsutum*, *T. aestivum, O. sativa*, *V. vinifera* and *Z. mays* was determined by constructing the phylogenetic tree using the protein sequences of putative NPR1 sequences of these crops. The study’s outcomes indicated that NPR1-like proteins could be categorized into three primary groups. The first cluster, identified as clade I (falling under the *AtNPR5* subfamily), comprises CaNPR4 and CaNPR5 (Yuan et al., 2007). The second cluster, known as clade II (part of the *AtNPR1/2/3* subfamily), consists of CaNPR1 and CaNPR3 (Feng et al., 2011). Finally, the third cluster, referred to as clade III (belonging to the *AtNPR4* subfamily), consists of CaNPR2 only (Fister et al., 2018; Figure 1).

**Figure 2:**
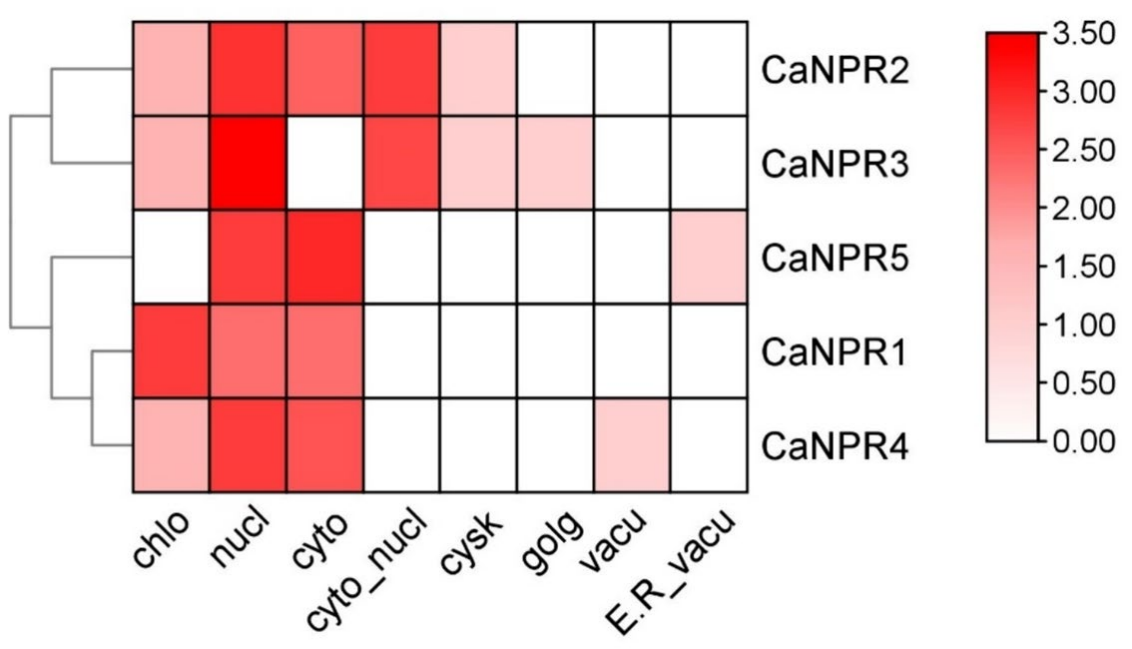
The subcellular localization prediction analysis of *CaNPR1* proteins indicated their predominant localization in chloroplasts, cytoplasm and nucleus. The analysis highlighted that the maximum number of proteins showed a red color, representing their localization in these cellular compartments.

The subcellular localization data for five genes denoted as *CaNPR1* through *CaNPR5*, reveals distinct patterns of presence in various cellular compartments. *CaNPR5* exhibits the least presence in the Chloroplast (Chlo), while *CaNPR1* displays the highest presence. In the Nucleus (Nucl), *CaNPR5* has the lowest presence, whereas *CaNPR3* exhibits the most substantial presence. Regarding the Cytoplasm (Cyto), *CaNPR5* showcases the highest presence, while *CaNPR3* has the least. In the Cytoplasmic Nucleus (Cyto_nucl), *CaNPR1, CaNPR4*, and *CaNPR5* share the minimal presence, with *CaNPR3* demonstrating the most prominent. For the Cytoskeleton (Cysk), all genes have similar low presence values, and in the Golgi apparatus (Golg) and Vacuole (Vacu), all genes show comparable minimal presence. Lastly, in the Endoplasmic Reticulum and Vacuole (E.R_vacu), all genes have a minimal presence, except for *CaNPR4*. These values provide insights into the diverse localization patterns of these genes within the cell (Figure 2).

### Conserved Cis-elements

The Cis-element analysis of the *CaNPR1* gene promoter regions had identified 36 cis-elements. In chili, *CaNPR1* genes were subjected to cis-element analysis, revealing various responsive elements, including 9 that are light-responsive elements, 1 stress-responsive element, 11 development and metabolism-responsive elements, 13 hormone-responsive elements, and 5 pathogen-responsive elements. The sensitivity to light observed in approximately 24.32% of these elements suggested the potential involvement of *CaNPR1* genes in responding to light stress. The presence of 2.70% of the cis-elements linked to stress responses also indicated a likely role for *CaNPR1* genes in stress-related activities. The study also identified that 29.72% of the cis-elements were associated with plant growth and metabolism, suggesting that *CaNPR1* genes might play a role in the growth and development of chili plants. Furthermore, the cis-elements responsive to hormones were discovered in 35.13% of cases, and biotic stress-responsive elements, such as the I-box.

**Figure 3:**
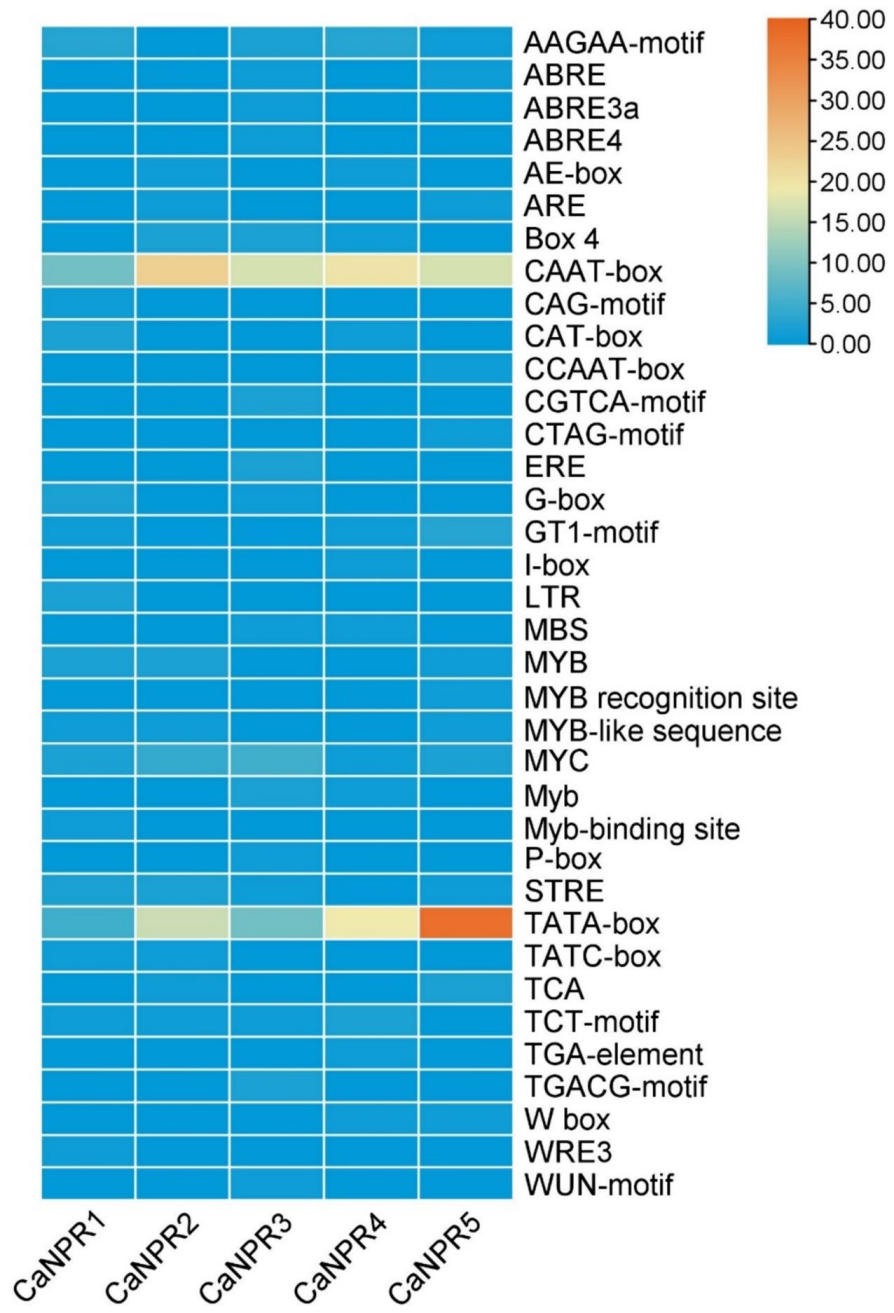
Examination of cis-regulatory elements in chili’s *CaNPR* gene promoters unveiled their involvement in diverse plant developmental processes. This analysis offered data on the occurrence of these elements within each *CaNPR* gene, and their spatial distribution across the promoters was also explored, providing insights into their arrangement.

TATC-box, TCA, and CAG-motif (cis-acting elements involved in pathogen and environmental stress responsiveness), were also found (Cheng et al., 2021). These elements represented intriguing targets for further study to understand hormone behavior under biotic stress conditions (Zeshan Haider et al., 2023). It was noted that all 5 *CaNPR1* genes contained the majority of the hormone-related and metabolism-responsive regions. Through the application of molecular breeding techniques, this analysis provided valuable insights into the potential roles of NPR1 genes in chili, facilitating the development of improved chili varieties (Supplementary Table S1 and Figure 3).

### Conserved motif analysis and domain prediction analysis of *CaNPRs*

**Figure 4:**
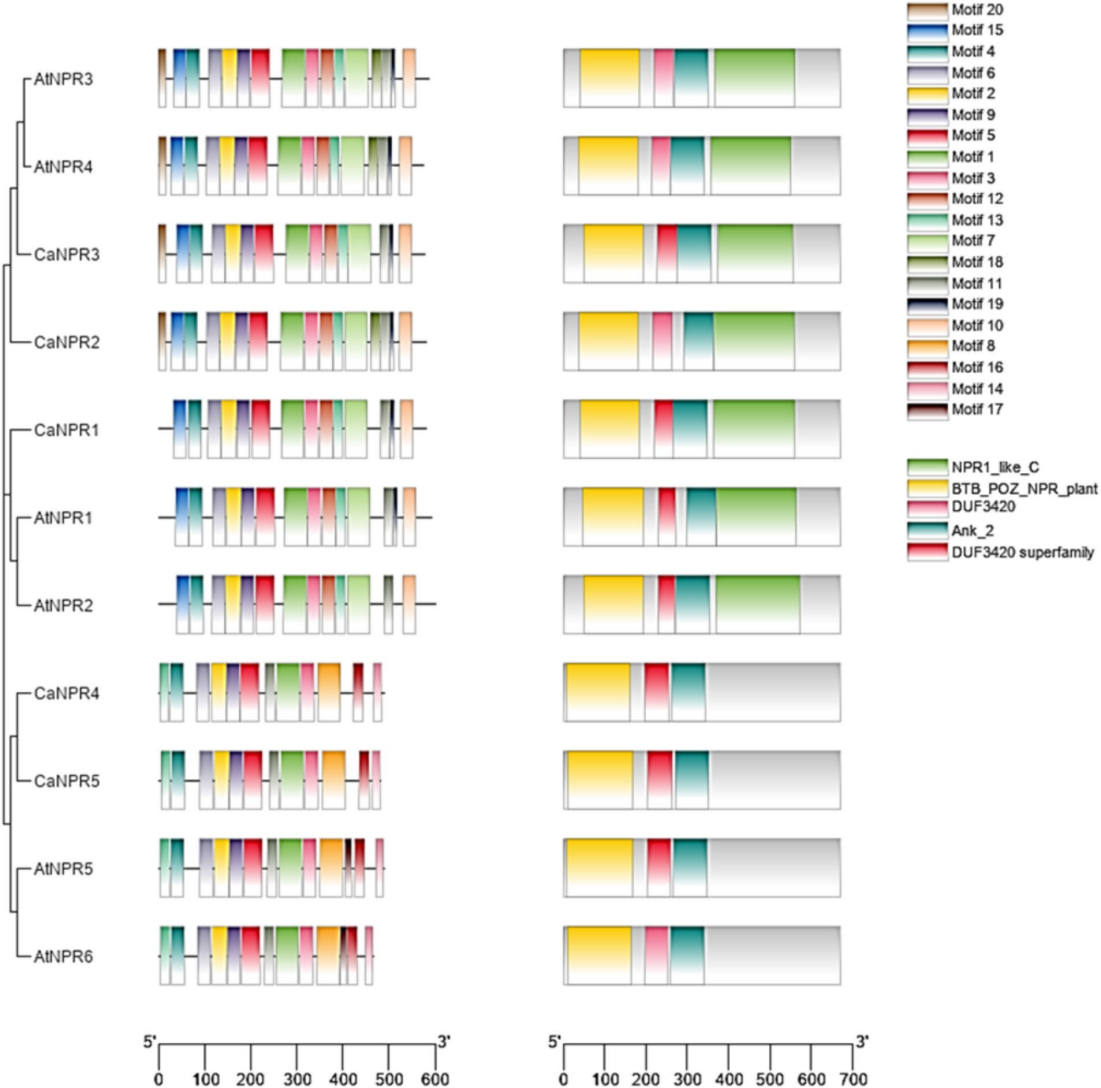
The analysis examined conserved domains and motifs in *CaNPR1* genes and their comparison with *A. thaliana* NPRs, along with the establishment of their phylogenetic relationships. This was visually represented through a color-coded bar graph, generated using MEME version 5.5.2, revealing 20 distinct motifs. By linking this graph to a phylogenetic tree, it offered valuable insights into the evolutionary patterns and functional connections among the CaNPR proteins.

The motif analysis of *NPR1* provided further confirmation by revealing a conserved domain and motif within the *C. annuum* genome. This analysis indicated that the NPR1-like genes in chili possessed domains like BTB and NPR-like C-terminal, along with Ankyrin repeats (Supplementary Table S2). Specifically, motif 1 (associated with Ankyrin repeats), 2 (Ankyrin repeats), 4 (BTB/POZ), 6 (NPR1/NIM1-like defense protein C-terminal) were present in all NPR1-like genes in chili (as depicted in Figure 2). Additionally, there were 16 motifs (numbered 20, 15, 4, 6, 2, 9, 5, 1, 3, 12, 13, 7, 18, 11, 19, and 10) conserved across all identified domains, encompassing NPR1-like-C, BTB-POZ-Plant, DUF3420, Ank-2, and the DUF3420 superfamily, which were found in *AtNPR3*, *AtNPR4, CaNPR3, CaNPR2, CaNPR1, AtNPR1*, and *AtNPR2*. On the other hand, there were 13 motifs (numbered 4, 6, 2, 9, 5, 1, 12, 16, 13, 7, 8, 17, and 19) conserved in the domains (BTB-POZ-Plant, DUF3420 superfamily, and Ank-2) identified in *CaNPR4, CaNPR5*, and *AtNPR5* (Zeshan Haider et al., 2023). For detailed information on the motif sequences and their functions in *CaNPR1* gene family (Supplementary Table S3 and Figure 4).

### Gene structure analysis

**Figure 5:**
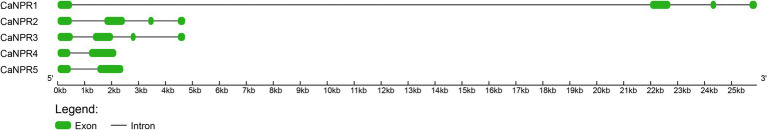
The exon-intron structures were depicted using shapes colored in yellow and black to represent the exons contained within the genes.

Based on predictions of gene structure, it was observed that *CaNPR1, CaNPR2,* and *CaNPR3* genes contained a total of 4 exons and 3 introns. In contrast, *CaNPR4* and *CaNPR5* had a simpler configuration with 2 exons and just 1 intron, indicating a more straightforward structure with interruptions in their coding sequences (Figure 5).

### MicroRNA analysis

All five *NPR1* genes were targeted by a total of 12 miRNAs. These 12 miRNAs are a part of 10 different miRNA groups. These miRNAs are between 20 and 22 amino acids long. Four families of miRNAs target *CaNPR3* (Can-miR6024, Can-miR3627, Can-miRN471, and Can-miRN475), making this the miRNA with the largest number of targeted sites. Four different families of miRNAs targeted *CaNPR5* (Can-miR319, Can-miRN17, Can-miRN453, and Can-miRN480). Only one extremely tiny miRNA, *CaNPR1* (Can-miRN462), and CaNPR2 (Can-miRN448), respectively, targeted these two *CaNPRs*. Although many miRNAs may target a single gene, miRNAs are selective and only target one gene. Most miRNAs prevent the cleavage of their targeted genes, whereas others prevent their translation. Two miRNAs prevent translation and eight miRNAs prevent cleavage together in 10 groupings (Wang M. et al., 2013). Several microRNAs, including miR6024, miR3627, miR471a, miR471b, miR319a, miR17, miR448, miR453a, miR453b, and miR462, play diverse roles in plant biology. miR6024 is associated with plant susceptibility to fungal pathogens, while miR3627 acts as a critical post-transcriptional regulator of gene expression by binding to complementary sequences in target mRNAs. miR471a and miR471b are involved in controlling both development and abiotic stress responses (Zeshan Haider et al., 2023). miR319a plays a role in plant development and the response to abiotic stress. miR17 is implicated in plant stem cell homeostasis and developmental programming. Additionally, miR448 contributes to the regulation of development and abiotic stress responses. Both miR453a and miR453b are involved in plant development, while miR462 plays a role in regulating highly conserved transcription factors, collectively contributing to the intricate network of post-transcriptional gene regulation in plants (Mazhar et al., 2023) described in Supplementary Table S4.

### Gene duplication and synteny analysis of *CaNPR1*

**Figure 6:**
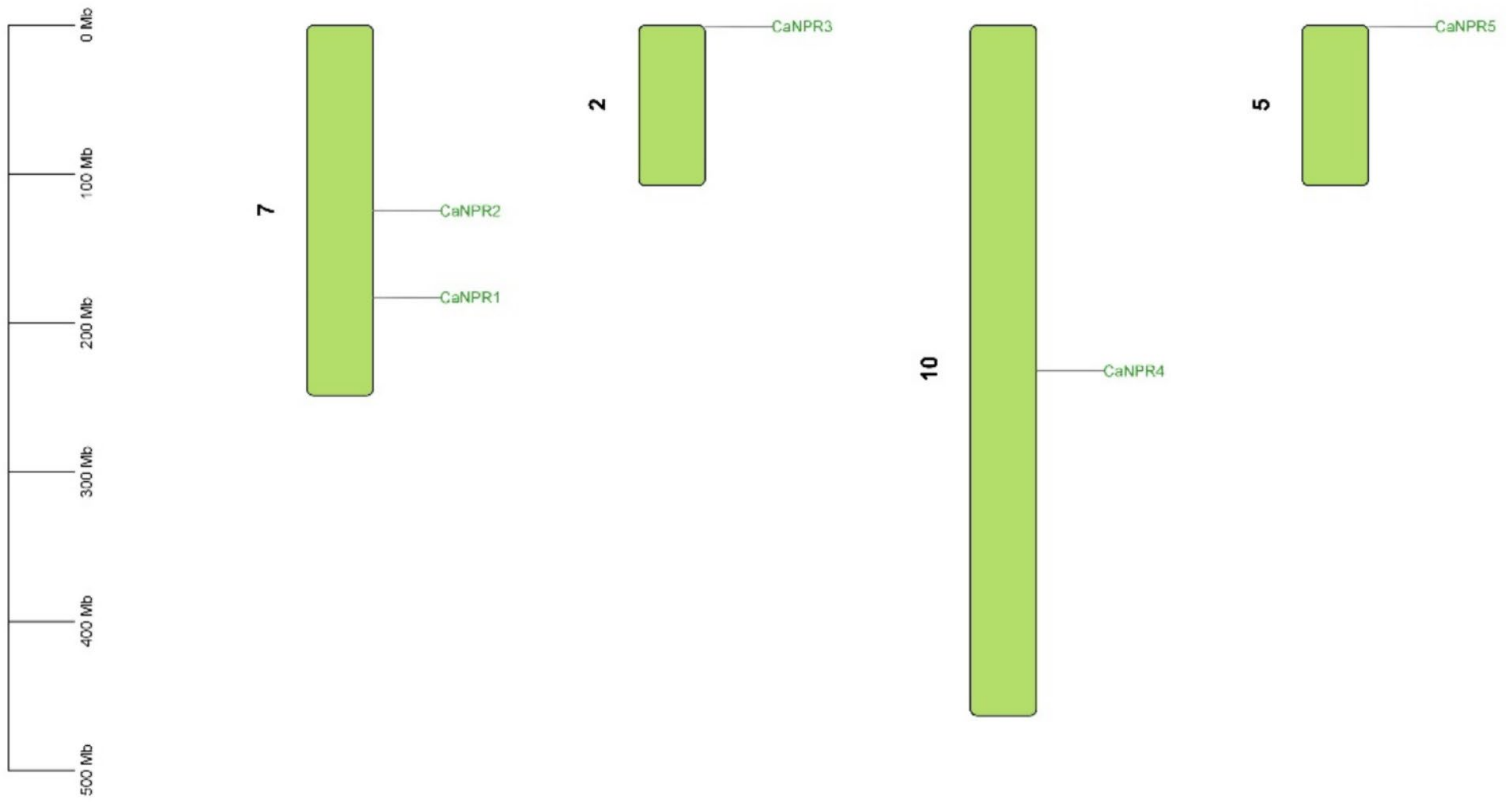
The chromosomal distribution of the *CaNPR* gene family in chili is illustrated in the figure. The figure’s green bars represent chili’s chromosomes, while the green lines indicate the functional relationships or co-regulation between *CaNPR* genes. This analysis provides insights into the spatial arrangement and potential interactions among *CaNPR* genes within the chili genome.

The expected chromosomal positions indicated that these genes were spread across different chromosomes. *NPR* genes were located on chromosome 7 (*CaNPR1, CaNPR2*), chromosome 2 (*CaNPR2*), chromosome 10 (*CaNPR4*), and chromosome 5 (*CaNPR5*) shown in Figure 6.

**Figure 7:**
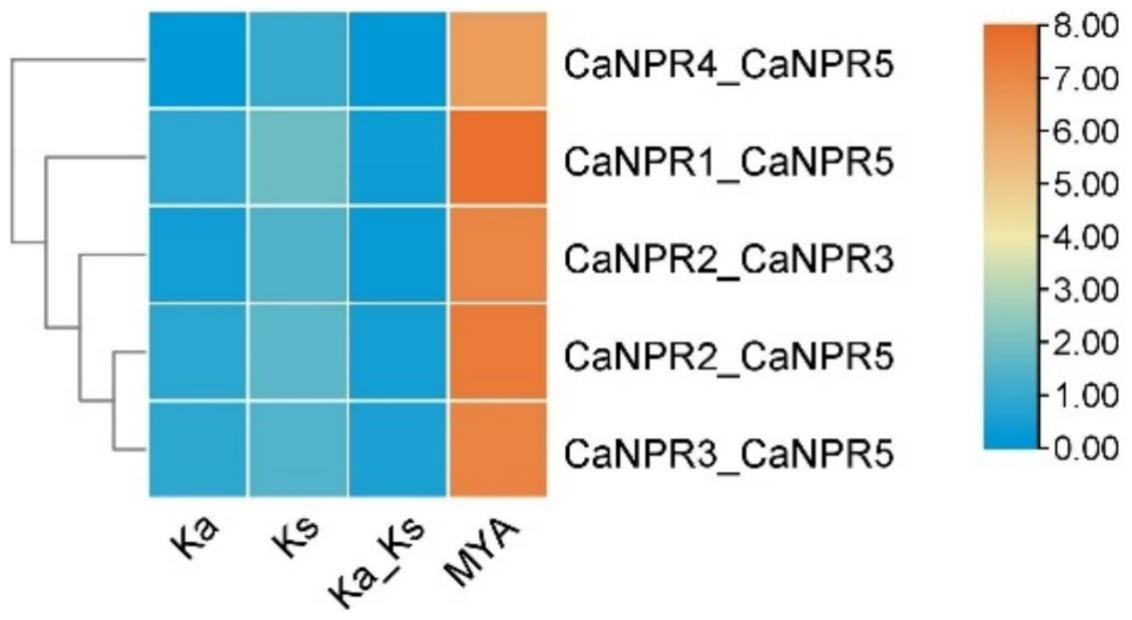
Ks (synonymous substitution rate) and Ka (nonsynonymous substitution rate) were computed through TB-tools. The rectangular rate (λ) for chili was assessed at 7.85 × 10*^−9^*. By employing the formula T = Ks/2λ, we determined the date of the duplication event. This analysis yields valuable information about the temporal and evolutionary aspects of gene duplications in chili.

According to the evolutionary relationship between chromosome location and gene family members, two gene pairs were found to be involved in gene duplication events. This ratio varied from 0.296 in the *CaNPR1/CaNPR5* pair, to 0.121 in the *CaNPR4/CaNPR5* pair. The predicted date for gene duplication of the paralogous genes *CaNPR1/CaNPR5* extended between 220.21 MYA to 82.13 in *CaNPR4/CaNPR5, respectively,* (Figure 7). Most of the paralogous gene pairs in chili had Ka/Ks ratios greater than 0.1 but less than 0.3, which suggests a possibility of significant functional deviation after the occurrence of duplication due to purifying selection (Islam et al., 2023).

**Figure 8:**
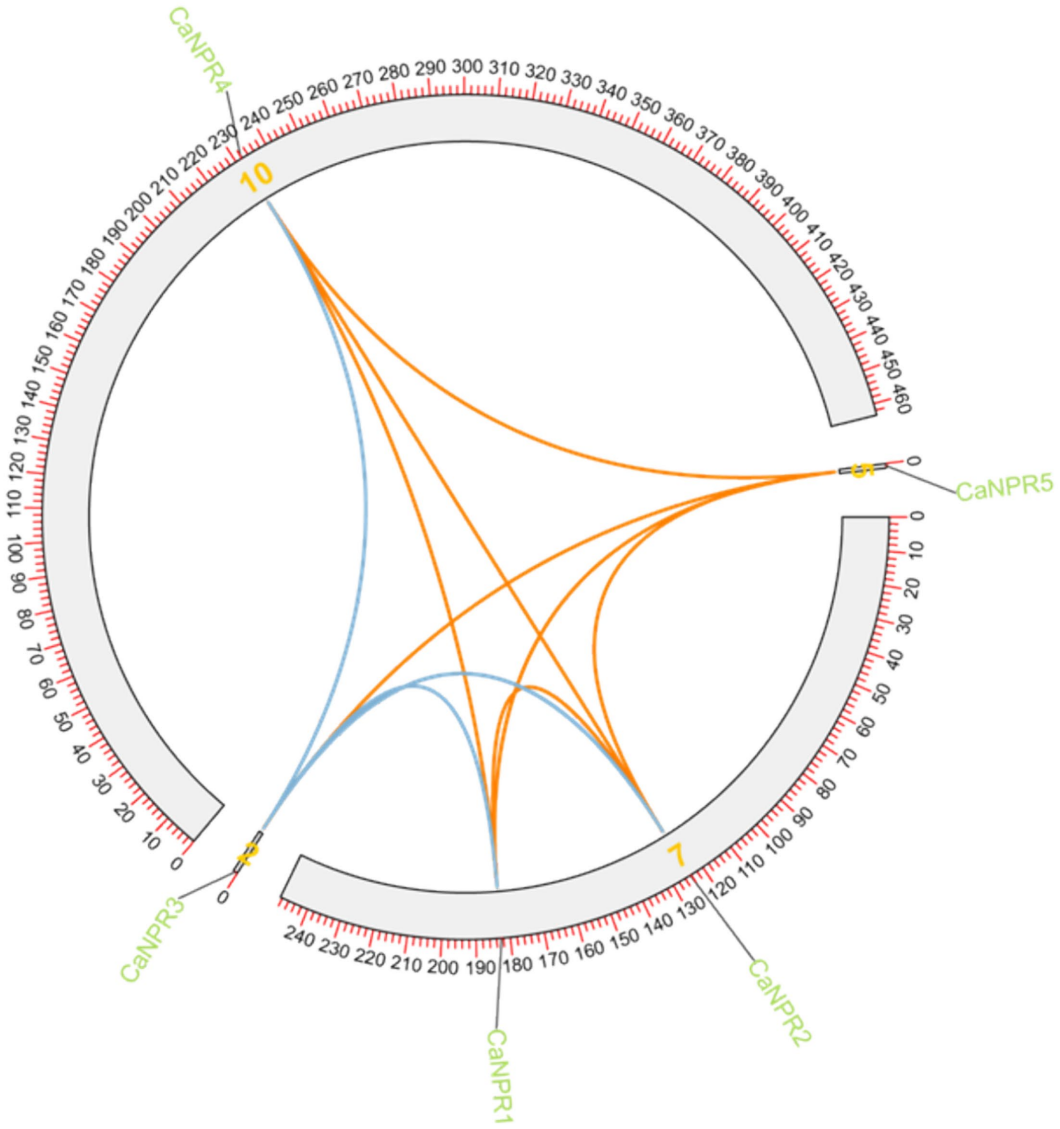
Single synteny illustrates how *CaNPR* genes are distributed across chromosomes, with lines connecting genes on separate chromosomes to indicate potential gene duplications.

Synteny analysis was performed to check out the gene duplication in different chromosomes of the chili. (*CaNPR2* and *CaNPR3*) & (*CaNPR4* and *CaNPR5*) were showing syntenic connection making overall two gene pairs. *CaNPR2* was located on chromosome 7 while its paired gene was located on chromosome 2 and *CaNPR4* was located on chromosome 10 whose paired gene *CaNPR5* was present on chromosome 5. These genes might suggest that they would have been the result of segmented duplication (Sami et al., 2024; Figure 8).

**Figure 9:**
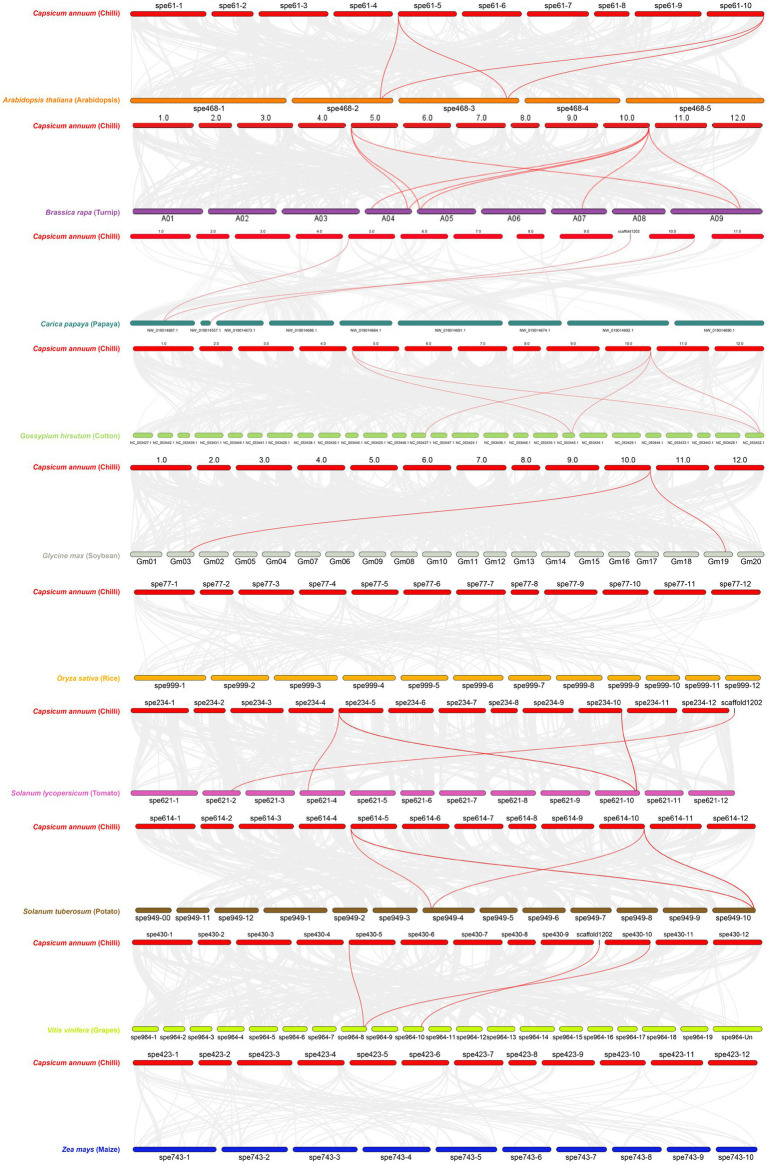
Dual Synteny plot of putative *NPR1* genes between *C. annuum* and 10 other plant species (red lines indicates the orthologous of *CaNPR1* in other plants).

Dual synteny between *C. annuum* and 10 other plants including *A. thaliana*, *S. tuberosum*, *S. lycopersicum*, *B. Rapa*, *C. papaya*, *G. max*, *G. hirsutum*, *O. sativa*, *V. vinifera,* and *Z. mays* showed variation in evolutionary gene duplication. One step MCScanX was adopted for generating files for the dual synteny analysis, the dual synteny maps were constructed using dual synteny plot for MCScanX. *Z. mays* and *O. sativa* were the two crops out of 10 that expressed zero duplication of orthologous *CaNPR* genes (Figure 9). All the remaining crops displayed syntenic relation but with slight variations in orthologues gene pairs. Some of the described *CaNPR* genes were making twice or thrice orthologous pairs with other plant species. It is suggested that proteins of connected genes may have similar functions because of the previous evolutionary linkage (Sami et al., 2024).

### Gene ontology

Gene ontology functionally annotated chili *NPR1* genes in biological processes, molecular functions and cellular components (Supplementary Table S5). Most vital biological processes i: e defense response to bacterium, incompatible interaction, regulation of salicylic acid-mediated signaling pathway, involvement in systemic acquired resistance, Plant organ development, involvement in JA-mediated signaling pathway, vitamin K1 biosynthetic process, amino acid biosynthesis, and tryptophan anabolism were mostly enriched. Likewise, the nucleus, plastid, and cytoplasm were found in the cellular compartment. Most common terms for molecular functions are antigen binding, pathogen-associated molecular pattern receptor activity, mitogen-activated protein kinase kinase kinase activity, kinase binding, jasmonoyl-valine synthetase activity, isochorismate synthetase activity, anthranilate synthetase activity, and transferase activity (Sami et al., 2024).

### Protein–protein interaction

**Figure 10:**
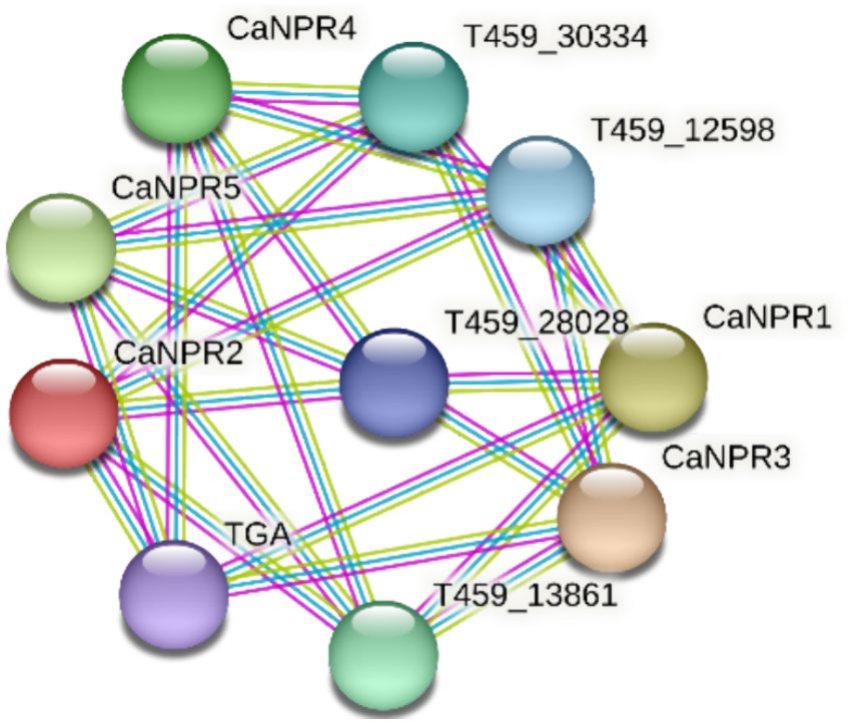
Visualization of the protein–protein interaction network among CaNPR proteins, illustrating their connectivity and interaction patterns.

The network comprises 10 proteins and 25 interactions, implying that each protein interacts with approximately 5 others. The average local clustering coefficient of 0 indicates no clustering within the CaNPR proteins, suggesting instead that they tend to interact with proteins outside this group (Sami et al., 2024; Figure 10).

### Virus titer quantification in both varieties

**Figure 11:**
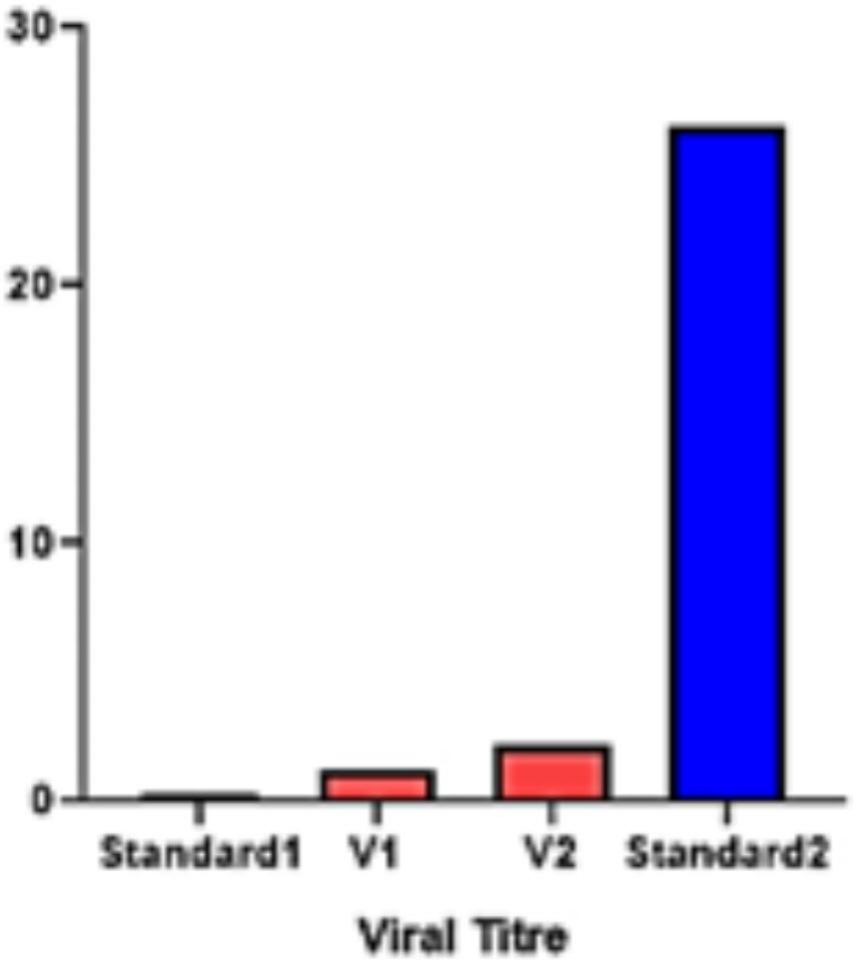
Virus titer quantification in both varieties; V1 (Resistant) and V2 (Susceptible) varieties.

To understand the role and vitality of all the identified *CaNPR* genes in chili under geminivirus stress in both V1 (resistant) and V2 (susceptible) varieties, the gene expression was quantified in chili leaf tissues by qRT-PCR analysis. For the absolute quantification of virus titer, separate tenfold serial dilutions of plasmid harboring the full length. Begomovirus was dissolved in an equal amount of healthy chili genomic DNA. Dilutions of plasmid were made in the range from 20 ng, 2 ng, 0.2 ng, 0.02 ng to 0.002 ng. These samples were then analyzed in triplicate. The mean threshold cycle (Ct) of the triplicates was used for the estimation of Begomovirus titer in both varieties of chili and was further compared with minimum and maximum standard plasmid dilutions. Both the varieties showed different levels of virus titer, V2 showed a higher quantity than V1 (Figure 11).

**Figure 12:**
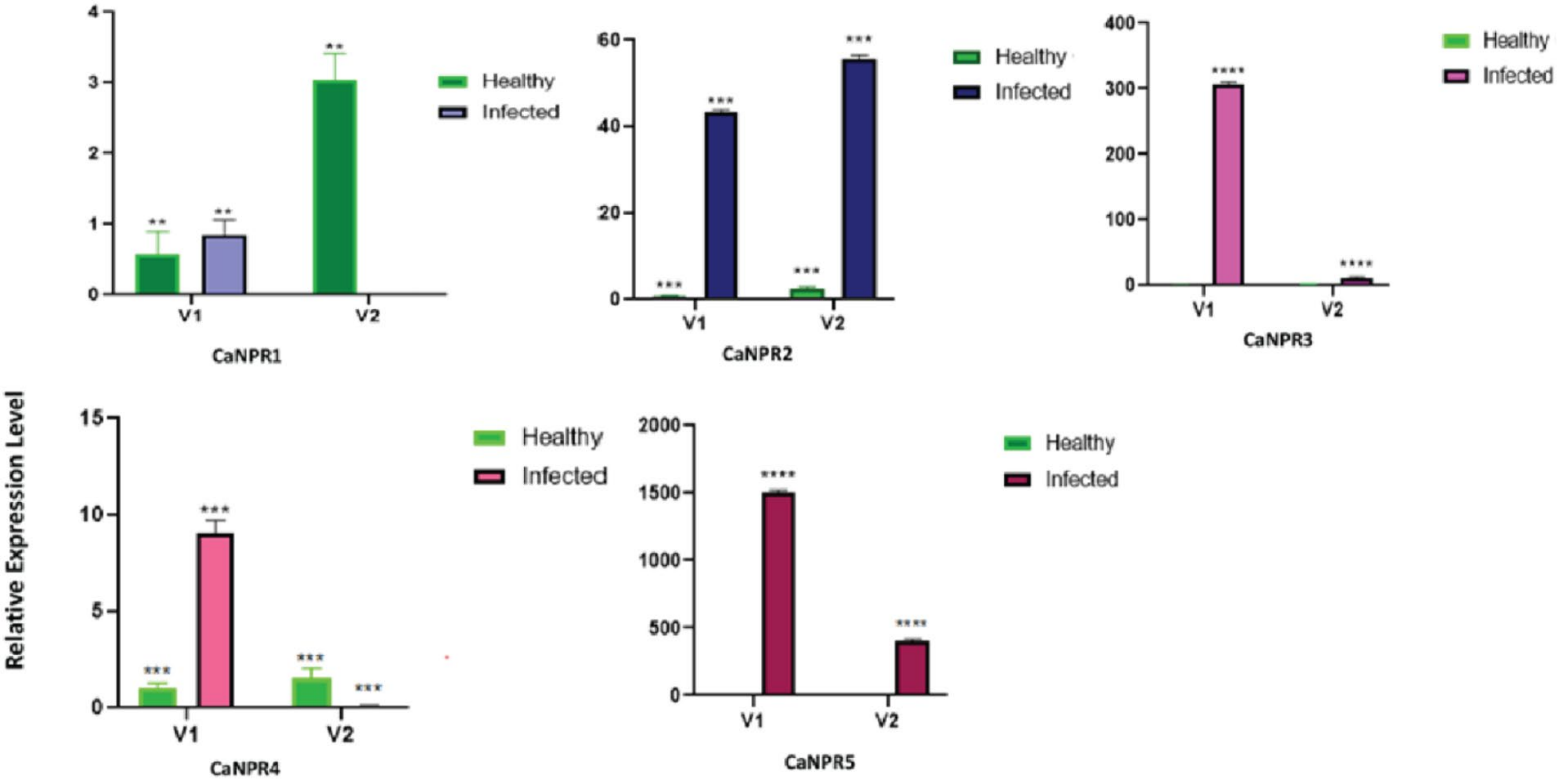
Relative expression of each identified *CaNPR1* genes in both varieties upon geminivirus infection.

The findings from the current investigations indicate a notably distinct response of NPR1 in both varieties. Each gene showed different expression against CLCuKV in both varieties on different viral titer. However, *CaNPR3* and *CaNPR4* exhibited higher expression in V1 and showed no or negligible expression in V2 this suggests that the expression of these two genes is inversely proportional to the virus titer. While the *CaNPR1* show inconsistent expression behaviour in V2 while its expression in V1 is almost same in healthy and infected plant (Figure 12).

## Discussion

Chili pepper (*C. annuum* L.) is a versatile crop in Pakistan, serving both as a vegetable and spice with significant economic value (Olatunji and Afolayan, 2018). However, its productivity is severely affected by various chili leaf curl geminiviruses (ChLCuGV), posing a considerable challenge for management. Successful control strategies rely on an integrated approach, (Mejía-Teniente et al., 2019), including cultural practices and novel techniques like genetically modified crops using RNA silencing, which target essential viral genome components. Despite these efforts, the issue persists. To combat this viral disease complex in an eco-friendly manner, a profound understanding of the molecular mechanisms behind chili pepper’s resistance to ChLCuGV infection is crucial. Plants utilize diverse defense mechanisms in their innate immune response against pathogens. Effector-triggered Immunity (ETI) exemplifies one such mechanism, where plants activate resistance (R) proteins that can induce apoptosis in infected cells, thereby halting further proliferation of the pathogen (Syukriani et al., 2015).

A comprehensive genome-wide investigation was undertaken to investigate *NPR1* genes within chili pepper. Five *CaNPR* genes were examined for their physicochemical properties to discern variations among these proteins within a particular clade. The analysis showed that all identified CaNPR1 proteins exhibit hydrophilic characteristics, indicating their affinity for water interaction and ability to carry electrical charges depending on pH levels. Additionally, the instability index analysis indicated that five of these proteins displayed features suggesting instability, while CaNPR3 appeared to be stable (Zeshan Haider et al., 2023).

A Comparative analysis of members within similar subgroups can provide valuable insights into their functional characteristics. In this study, we identified 100 NPR1 proteins and categorized them into three clades based on their sequence structures and evolutionary relationships with *Arabidopsis* (Zeshan Haider et al., 2023). Through phylogenetic analysis, we identified five CaNPR1 proteins. Two of these CaNPR4 and CaNPR5 were located in clade I alongside *Arabidopsis* proteins AtNPR5 and AtNPR6. CaNPR1 and CaNPR3 were positioned in clade II with AtNPR1, AtNPR2, and AtNPR3. Clade III featured just one CaNPR2, which aligned with AtNPR4. These findings suggest that CaNPR4 and CaNPR5 proteins in their respective subgroups may have roles similar to those of AtNPR6 and AtNPR6 in *Arabidopsis*. In contrast, CaNPR4 and CaNPR5 proteins may function similarly to those of AtNPR1, AtNPR2, and AtNPR3 proteins, and CaNPR2 may serve function analogously to AtNPR4 proteins (Yuan et al., 2007).

The subcellular localization investigation revealed that *CaNPR1* proteins were distributed across various organelles, including the chloroplast, mitochondria, cytoplasm, cytosol, endoplasmic reticulum, nucleus, and plasma membrane. A notable portion of these proteins, precisely 39.51, 25.31, and 14.81%, were, respectively, found in the nucleus, cytoplasm, and chloroplast. This indicates that *CaNPR1* proteins might play important roles within these specific organelles. Cis-regulatory elements commonly found in the gene promoter region are crucial for controlling gene expression at the transcriptional level. The study identified that 29.72% of the cis-elements were associated with plant growth and metabolism, suggesting that *CaNPR1* genes might have played a role in the growth and development of chili plants. Additionally, the second-largest group, comprising approximately 24.32% of these elements, indicated the potential involvement of *CaNPR1* genes in responding to light-induced stress. Furthermore, *CaNPR1* contained various motifs responsible for different responses, such as the CGTCA-motif and TGACG-motif for the MeJA response, the TCA-element for the SA response, the GARE-motif, TATC-box, and P-box for the GA response, and the ABRE, TGA-element for the Auxin response. Moreover, cis-elements responsive to hormones were discovered in 13.88% of cases, and biotic stress-responsive elements, such as the I-box, TATC-box, TCA, and CAG-motif (cis-acting elements involved in pathogen and environmental stress responsiveness), were also identified. These elements represented intriguing targets for further study to understand hormone behavior under biotic stress conditions (Zeshan Haider et al., 2023).

Past studies have indicated the significance of how exons and introns were positioned within gene families for evolution (Zhao et al., 2022). The examination in this study of gene structure and motifs showed that the arrangement of exons, introns, and motifs within members of the same group and clade aligned with the phylogenetic tree’s pattern (Wei et al., 2022). Each *NPR1-like* gene contained both exons and introns. It’s noteworthy that, in line with plant characteristics, the motifs within the *CaNPR1* subgroups appeared to be more conserved. Conserved motifs in genes were significant because they typically indicated essential functional elements for the gene’s role. Their preservation suggested evolutionary importance and shared functionality among related genes.

MicroRNAs (miRNAs) were pivotal regulatory molecules in plants, exerting a substantial influence on various biological processes, encompassing plant growth, development, and responses to both biotic and abiotic stress (Ding et al., 2020). They were notably conserved and had specific functional roles (Millar et al., 2019). The investigation revealed that *CaNPR1* was associated with several microRNAs, including miR6024, miR3627, miR471a, miR471b, miR319a, miR17, miR448, miR453a, miR453b, and miR462, each contributing to diverse aspects of plant biology. For instance, miR6024 was linked to plant susceptibility to fungal pathogens, while miR3627 functioned as a crucial post-transcriptional gene expression regulator by binding to complementary sequences in target mRNAs. Both miR471a and miR471b contribute to developmental control and responses to abiotic stress. miR319a is involved in plant development and its response to abiotic stress, while miR17 maintains plant stem cell homeostasis and developmental programming. Furthermore, miR448 regulates development and responses to abiotic stress. Notably, miR453a and miR453b had roles in plant development, while miR462 played a part in regulating highly conserved transcription factors, collectively participating in the intricate network of post-transcriptional gene regulation in plants. These findings suggested that miRNAs had played a highly significant role in managing stress, promoting growth and development, and responding to biotic stress in chili plants (Zeshan Haider et al., 2023).

The anticipated chromosomal positions suggested a dispersed gene distribution, aiding in understanding gene family evolution and genome-wide distribution, supporting comparative genomics and phylogenetic investigations. Synteny analysis revealed gene duplications in different chili chromosomes, possibly resulting from segmental duplications in the chili genome. Comparative analysis with 10 other plant species, including *A. thaliana, S. tuberosum, S. lycopersicum, B. Rapa, C. papaya, G. max, G. hirsutum, O. sativa, V. vinifera, and Z. mays*, showed variations in the evolutionary gene duplications. Among these, *Z. mays* and *O. sativa* displayed no orthologous *CaNPR* gene duplications, while other crops exhibited synteny relationships with slight variations in orthologous gene pairs. Some *CaNPR* genes formed multiple orthologous pairs with other plant species, suggesting potential functional similarities due to shared evolutionary history. Based on the evolutionary relationship between chromosome locations and gene family members, two gene pairs were identified as being involved in gene duplication events. Most of the paralogous gene pairs in chili had Ka/Ks ratios above 0.1 but below 0.3, indicating the possibility of significant functional divergence after duplication, likely influenced by purifying selection (Islam et al., 2023).

The functional annotation of chili *NPR1* genes covered biological processes, molecular functions, and cellular components. Key processes included defense responses, signal pathway regulation, and organ development, with cellular locations primarily in the nucleus, plastids, and cytoplasm. Molecular functions identified included antigen binding, kinase activity, and synthesis-related activities. Protein–protein interaction revealed that an average local clustering coefficient of 0 indicates a lack of clustering among the CaNPR proteins, implying that they do not form tightly interconnected subgroups. Instead, they tend to interact with proteins outside their immediate group, suggesting a more dispersed network of interactions (Jamsari et al., 2019).

In this study, the *NPR1* gene family, renowned for its involvement in plant defense against biotic stress and salicylic acid production, was uncovered in the *C. annuum* genome. The research thoroughly investigated the attributes of five *CaNPR1*-like genes to comprehend their roles in *C. annuum*. Identification of these genes was based on their BTB and Chorismate binding domains, and their count differed from that in *Arabidopsis*. To predict their functions, the study quantified the expression of *CaNPR1* genes in chili pepper leaf tissues exposed to geminivirus stress. Two chili pepper varieties were tested for changes in gene expression upon geminivirus infection. While both varieties showed resistance (V1) and susceptibility (V2) to the inoculated geminivirus. The virus titer measurement indicated that V2 had a higher titer than V1. In terms of gene expression, *CaNPR1* displayed elevated levels in inoculated V2, whereas the other genes exhibited higher expression in inoculated V1. These results imply that *CaNPR1* might confer prolonged resistance, while the remaining genes are active during the early stages of infection (Son et al., 2021). Nonetheless, the study underscores the collective contribution of all genes to geminivirus resistance (Mejía-Teniente et al., 2019). While this study offers valuable insights into the NPR1 gene family in chili pepper, it relies heavily on in-silico data with limited *in-vivo* validation. Future research should focus on experimental confirmation of gene functions under diverse biotic and abiotic stresses. Additionally, exploring genetic diversity across chili pepper cultivars and conducting broader comparative studies with other species would enhance the applicability and depth of our findings. The findings of this study offer new perspectives on the functional versatility and evolutionary aspects of the *CaNPR1* gene family in plants. These revelations will be a valuable resource for forthcoming studies, simplifying the exploration of their functions and gene cloning. The thorough genome-wide identification and characterization, as conducted in this research, will pave the way for more in-depth investigations in the future.

## Conclusion

This study identified five *CaNPR* genes in chili, and our structural analysis indicated variations in the number of introns and exons, ranging from one to four, within these genes. The presence of cis-regulatory elements in the gene promoters suggested their involvement in chili’s response to biotic stress, including factors like light responsiveness, development, hormones, and certain biotic stressors. MicroRNAs (miRNAs) were pivotal regulatory molecules in plants, exerting a substantial influence on various biological processes, encompassing plant growth, development, and responses to both biotic and abiotic stresses. Upon inoculation with cotton leaf curl Khokhran virus, chili plant growth was adversely affected, resulting in stunted development, fibrous roots, and visible virus symptoms. Our qRT-PCR results suggested that *CaNPR1* potentially imparts prolonged resistance against geminivirus and contributes to chili plant defense mechanisms, whereas the other genes become active during the early infection stages. Nevertheless, further research, encompassing gene cloning and functional analysis, is necessary to validate the roles of these genes in various physiological and biological processes. This in-silico analysis enriches our genome-wide understanding of chili *CaNPR* genes.

## Data availability statement

I affirm that all necessary data and permissions have been provided for this study. Any interested researchers can access the required data to support the findings and conclusions of this article. For publicly archived datasets, hyperlinks are provided in this manuscript in appropriate place for convenience.

## Author contributions

QI: Conceptualization, Methodology, Writing – original draft. AS: Investigation, Supervision, Writing – review & editing, Writing – original draft. MZ: Project administration, Visualization, Writing – review & editing, Writing – original draft. AA: Formal analysis, Software, Writing – review & editing. MS: Supervision, Writing – original draft, Writing – review & editing. QA: Supervision, Writing – review & editing. AB: Validation, Visualization, Writing – review & editing. MH: Data curation, Formal Analysis, Writing – review & editing. DA: Conceptualization, Validation, Visualization, Writing – review & editing. SA: Investigation, Writing – review & editing. MI: Investigation, Writing – review & editing. MM: Supervision, Visualization, Writing – review & editing.

## Footnotes

^1^ https://plants.ensembl.org/Capsicum_annuum/Tools/Blast

^2^ https://www.ncbi.nlm.nih.gov/

^3^ https://web.expasy.org/protparam/

^4^ https://plants.ensembl.org/

^5^ https://wolfpsort.hgc.jp/

^7^ http://bioinformatics.psb.ugent.be/webtools/plantcare/html/

^8^ https://meme-suite.org/meme/tools/meme

^9^ https://www.pmiren.com/

^10^ https://www.zhaolab.org/psRNATarget/

^11^ https://cytoscape.org/

^12^ https://string-db.org/

^13^ https://www.ncbi.nlm.nih.gov/tools/primer-blast/
